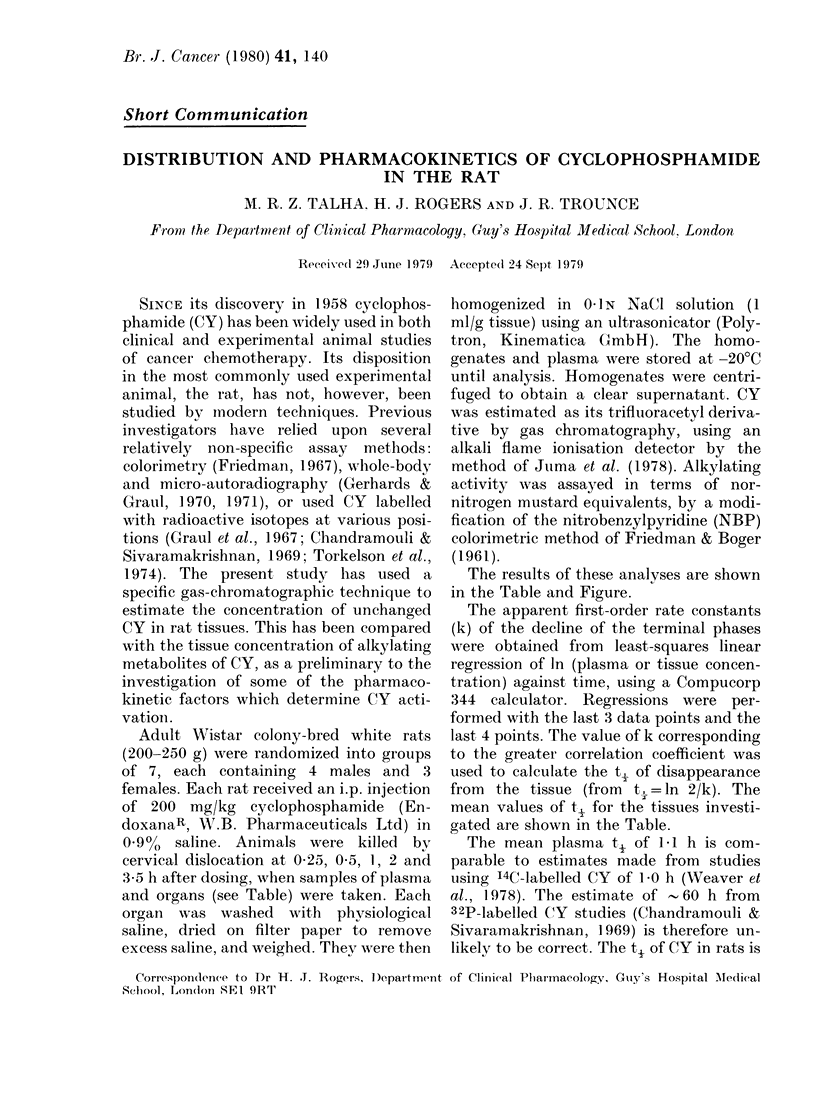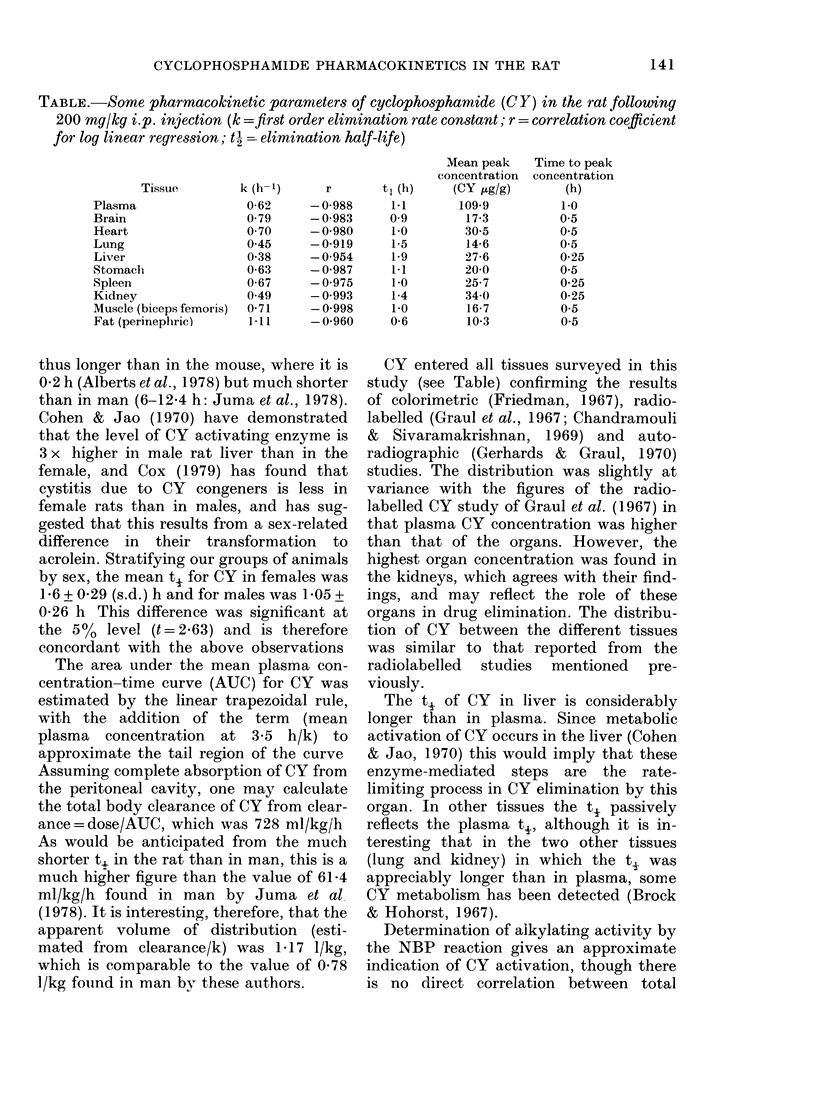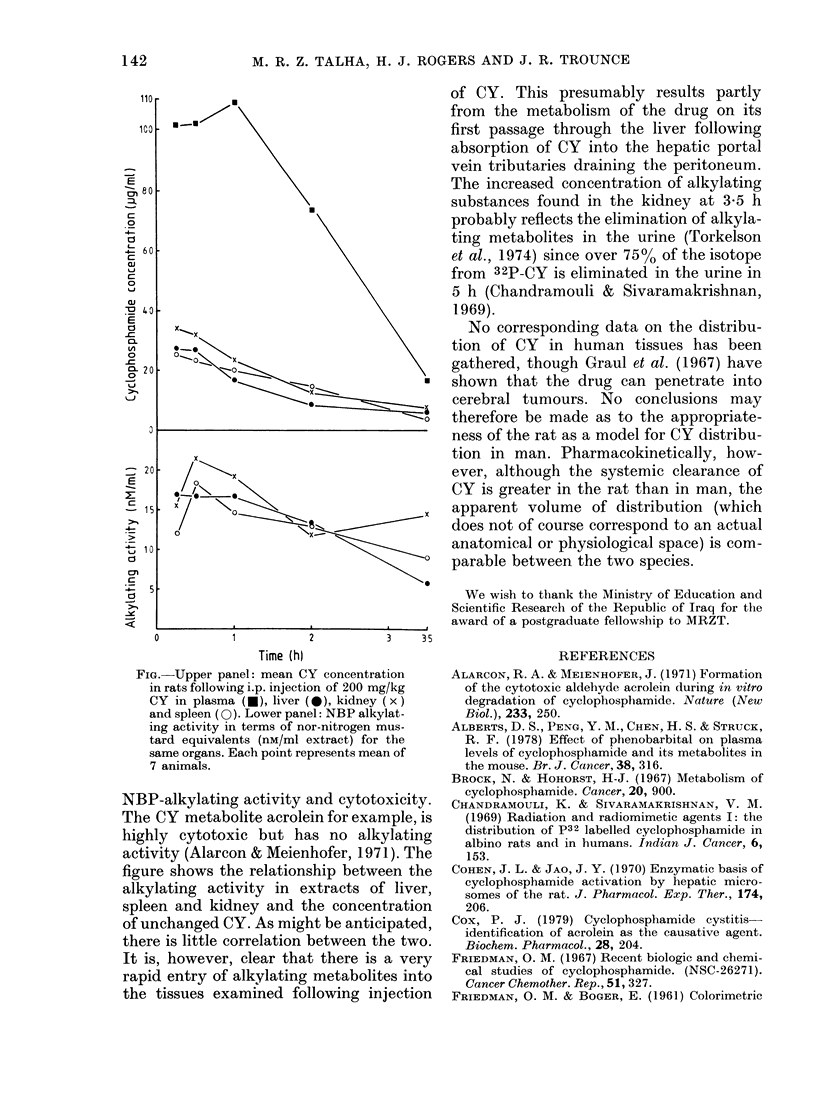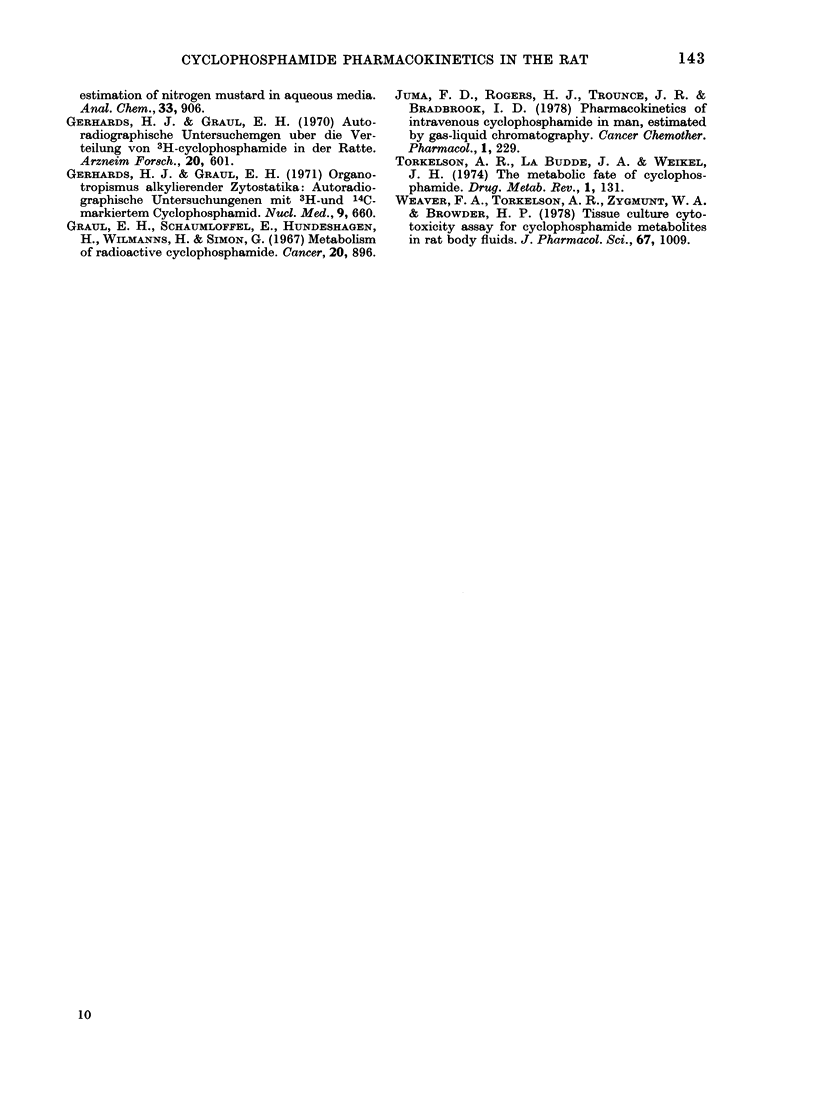# Distribution and pharmacokinetics of cyclophosphamide in the rat.

**DOI:** 10.1038/bjc.1980.17

**Published:** 1980-01

**Authors:** M. R. Talha, H. J. Rogers, J. R. Trounce


*Br. J. Cancer* (1980) **41**, 140

***Short Communication***

### DISTRIBUTION AND PHARMACOKINETICS OF CYCLOPHOSPHAMIDE IN THE RAT

M. R. Z. TALHA, H. J. ROGERS AND J. R. TROUNCE

*From the Department of Clinical Pharmacology, Guy's Hospital Medical School, London*

Received 29 June 1979 Accepted 24 Sept 1979

SINCE its discovery in 1958 cyclophosphamide (CY) has been widely used in both clinical and experimental animal studies of cancer chemotherapy. Its disposition in the most commonly used experimental animal, the rat, has not, however, been studied by modern techniques. Previous investigators have relied upon several relatively non-specific assay methods: colorimetry (Friedman, 1967), whole-body and micro-autoradiography (Gerhards & Graul, 1970, 1971), or used CY labelled with radioactive isotopes at various positions (Graul *et al.*, 1967; Chandramouli & Sivaramakrishnan, 1969; Torkelson *et al.*, 1974). The present study has used a specific gas-chromatographic technique to estimate the concentration of unchanged CY in rat tissues. This has been compared with the tissue concentration of alkylating metabolites of CY, as a preliminary to the investigation of some of the pharmacokinetic factors which determine CY activation.

Adult Wistar colony-bred white rats (200–250 g) were randomized into groups of 7, each containing 4 males and 3 females. Each rat received an i.p. injection of 200 mg/kg cyclophosphamide (Endoxana$^R$, W.B. Pharmaceuticals Ltd) in 0·9% saline. Animals were killed by cervical dislocation at 0·25, 0·5, 1, 2 and 3·5 h after dosing, when samples of plasma and organs (see Table) were taken. Each organ was washed with physiological saline, dried on filter paper to remove excess saline, and weighed. They were then homogenized in 0·1N NaCl solution (1 ml/g tissue) using an ultrasonicator (Polytron, Kinematica GmbH). The homogenates and plasma were stored at −20°C until analysis. Homogenates were centrifuged to obtain a clear supernatant. CY was estimated as its trifluoracetyl derivative by gas chromatography, using an alkali flame ionisation detector by the method of Juma *et al.* (1978). Alkylating activity was assayed in terms of nor-nitrogen mustard equivalents, by a modification of the nitrobenzylpyridine (NBP) colorimetric method of Friedman & Boger (1961).

The results of these analyses are shown in the Table and Figure.

The apparent first-order rate constants (k) of the decline of the terminal phases were obtained from least-squares linear regression of ln (plasma or tissue concentration) against time, using a Compucorp 344 calculator. Regressions were performed with the last 3 data points and the last 4 points. The value of k corresponding to the greater correlation coefficient was used to calculate the $t_{\frac{1}{2}}$ of disappearance from the tissue (from $t_{\frac{1}{2}} = \ln 2/k$). The mean values of $t_{\frac{1}{2}}$ for the tissues investigated are shown in the Table.

The mean plasma $t_{\frac{1}{2}}$ of 1·1 h is comparable to estimates made from studies using $^{14}$C-labelled CY of 1·0 h (Weaver *et al.*, 1978). The estimate of ~60 h from $^{32}$P-labelled CY studies (Chandramouli & Sivaramakrishnan, 1969) is therefore unlikely to be correct. The $t_{\frac{1}{2}}$ of CY in rats is

Correspondence to Dr H. J. Rogers, Department of Clinical Pharmacology, Guy's Hospital Medical School, London SE1 9RT

TABLE.—*Some pharmacokinetic parameters of cyclophosphamide (CY) in the rat following 200 mg/kg i.p. injection (k = first order elimination rate constant; r = correlation coefficient for log linear regression; $t\frac{1}{2}$ = elimination half-life)*

| Tissue | k ($h^{-1}$) | r | $t_{\frac{1}{2}}$ (h) | Mean peak concentration (CY μg/g) | Time to peak concentration (h) |
|---|---|---|---|---|---|
| Plasma | 0·62 | −0·988 | 1·1 | 109·9 | 1·0 |
| Brain | 0·79 | −0·983 | 0·9 | 17·3 | 0·5 |
| Heart | 0·70 | −0·980 | 1·0 | 30·5 | 0·5 |
| Lung | 0·45 | −0·919 | 1·5 | 14·6 | 0·5 |
| Liver | 0·38 | −0·954 | 1·9 | 27·6 | 0·25 |
| Stomach | 0·63 | −0·987 | 1·1 | 20·0 | 0·5 |
| Spleen | 0·67 | −0·975 | 1·0 | 25·7 | 0·25 |
| Kidney | 0·49 | −0·993 | 1·4 | 34·0 | 0·25 |
| Muscle (biceps femoris) | 0·71 | −0·998 | 1·0 | 16·7 | 0·5 |
| Fat (perinephric) | 1·11 | −0·960 | 0·6 | 10·3 | 0·5 |

thus longer than in the mouse, where it is 0·2 h (Alberts *et al.*, 1978) but much shorter than in man (6–12·4 h: Juma *et al.*, 1978). Cohen & Jao (1970) have demonstrated that the level of CY activating enzyme is 3× higher in male rat liver than in the female, and Cox (1979) has found that cystitis due to CY congeners is less in female rats than in males, and has suggested that this results from a sex-related difference in their transformation to acrolein. Stratifying our groups of animals by sex, the mean $t_{\frac{1}{2}}$ for CY in females was $1·6 \pm 0·29$ (s.d.) h and for males was $1·05 \pm 0·26$ h This difference was significant at the 5% level ($t = 2·63$) and is therefore concordant with the above observations

The area under the mean plasma concentration–time curve (AUC) for CY was estimated by the linear trapezoidal rule, with the addition of the term (mean plasma concentration at 3·5 h/k) to approximate the tail region of the curve Assuming complete absorption of CY from the peritoneal cavity, one may calculate the total body clearance of CY from clearance = dose/AUC, which was 728 ml/kg/h As would be anticipated from the much shorter $t_{\frac{1}{2}}$ in the rat than in man, this is a much higher figure than the value of 61·4 ml/kg/h found in man by Juma *et al.* (1978). It is interesting, therefore, that the apparent volume of distribution (estimated from clearance/k) was 1·17 l/kg, which is comparable to the value of 0·78 l/kg found in man by these authors.

CY entered all tissues surveyed in this study (see Table) confirming the results of colorimetric (Friedman, 1967), radiolabelled (Graul *et al.*, 1967; Chandramouli & Sivaramakrishnan, 1969) and autoradiographic (Gerhards & Graul, 1970) studies. The distribution was slightly at variance with the figures of the radiolabelled CY study of Graul *et al.* (1967) in that plasma CY concentration was higher than that of the organs. However, the highest organ concentration was found in the kidneys, which agrees with their findings, and may reflect the role of these organs in drug elimination. The distribution of CY between the different tissues was similar to that reported from the radiolabelled studies mentioned previously.

The $t_{\frac{1}{2}}$ of CY in liver is considerably longer than in plasma. Since metabolic activation of CY occurs in the liver (Cohen & Jao, 1970) this would imply that these enzyme-mediated steps are the rate-limiting process in CY elimination by this organ. In other tissues the $t_{\frac{1}{2}}$ passively reflects the plasma $t_{\frac{1}{2}}$, although it is interesting that in the two other tissues (lung and kidney) in which the $t_{\frac{1}{2}}$ was appreciably longer than in plasma, some CY metabolism has been detected (Brock & Hohorst, 1967).

Determination of alkylating activity by the NBP reaction gives an approximate indication of CY activation, though there is no direct correlation between total

FIG.—Upper panel: mean CY concentration in rats following i.p. injection of 200 mg/kg CY in plasma (■), liver (●), kidney (×) and spleen (○). Lower panel: NBP alkylating activity in terms of nor-nitrogen mustard equivalents (nM/ml extract) for the same organs. Each point represents mean of 7 animals.

NBP-alkylating activity and cytotoxicity. The CY metabolite acrolein for example, is highly cytotoxic but has no alkylating activity (Alarcon & Meienhofer, 1971). The figure shows the relationship between the alkylating activity in extracts of liver, spleen and kidney and the concentration of unchanged CY. As might be anticipated, there is little correlation between the two. It is, however, clear that there is a very rapid entry of alkylating metabolites into the tissues examined following injection of CY. This presumably results partly from the metabolism of the drug on its first passage through the liver following absorption of CY into the hepatic portal vein tributaries draining the peritoneum. The increased concentration of alkylating substances found in the kidney at 3·5 h probably reflects the elimination of alkylating metabolites in the urine (Torkelson *et al.*, 1974) since over 75% of the isotope from $^{32}$P-CY is eliminated in the urine in 5 h (Chandramouli & Sivaramakrishnan, 1969).

No corresponding data on the distribution of CY in human tissues has been gathered, though Graul *et al.* (1967) have shown that the drug can penetrate into cerebral tumours. No conclusions may therefore be made as to the appropriateness of the rat as a model for CY distribution in man. Pharmacokinetically, however, although the systemic clearance of CY is greater in the rat than in man, the apparent volume of distribution (which does not of course correspond to an actual anatomical or physiological space) is comparable between the two species.

We wish to thank the Ministry of Education and Scientific Research of the Republic of Iraq for the award of a postgraduate fellowship to MRZT.

REFERENCES

ALARCON, R. A. & MEIENHOFER, J. (1971) Formation of the cytotoxic aldehyde acrolein during *in vitro* degradation of cyclophosphamide. *Nature* (*New Biol.*), **233**, 250.

ALBERTS, D. S., PENG, Y. M., CHEN, H. S. & STRUCK, R. F. (1978) Effect of phenobarbital on plasma levels of cyclophosphamide and its metabolites in the mouse. *Br. J. Cancer*, **38**, 316.

BROCK, N. & HOHORST, H-J. (1967) Metabolism of cyclophosphamide. *Cancer*, **20**, 900.

CHANDRAMOULI, K. & SIVARAMAKRISHNAN, V. M. (1969) Radiation and radiomimetic agents I: the distribution of $P^{32}$ labelled cyclophosphamide in albino rats and in humans. *Indian J. Cancer*, **6**, 153.

COHEN, J. L. & JAO, J. Y. (1970) Enzymatic basis of cyclophosphamide activation by hepatic microsomes of the rat. *J. Pharmacol. Exp. Ther.*, **174**, 206.

COX, P. J. (1979) Cyclophosphamide cystitis—identification of acrolein as the causative agent. *Biochem. Pharmacol.*, **28**, 204.

FRIEDMAN, O. M. (1967) Recent biologic and chemical studies of cyclophosphamide. (NSC-26271). *Cancer Chemother. Rep.*, **51**, 327.

FRIEDMAN, O. M. & BOGER, E. (1961) Colorimetric estimation of nitrogen mustard in aqueous media. *Anal. Chem.*, **33**, 906.

GERHARDS, H. J. & GRAUL, E. H. (1970) Autoradiographische Untersuchemgen uber die Verteilung von $^3$H-cyclophosphamide in der Ratte. *Arzneim Forsch.*, **20**, 601.

GERHARDS, H. J. & GRAUL, E. H. (1971) Organotropismus alkylierender Zytostatika: Autoradiographische Untersuchungenen mit $^3$H-und $^{14}$C-markiertem Cyclophosphamid. *Nucl. Med.*, **9**, 660.

GRAUL, E. H., SCHAUMLOFFEL, E., HUNDESHAGEN, H., WILMANNS, H. & SIMON, G. (1967) Metabolism of radioactive cyclophosphamide. *Cancer*, **20**, 896.

JUMA, F. D., ROGERS, H. J., TROUNCE, J. R. & BRADBROOK, I. D. (1978) Pharmacokinetics of intravenous cyclophosphamide in man, estimated by gas-liquid chromatography. *Cancer Chemother. Pharmacol.*, **1**, 229.

TORKELSON, A. R., LA BUDDE, J. A. & WEIKEL, J. H. (1974) The metabolic fate of cyclophosphamide. *Drug. Metab. Rev.*, **1**, 131.

WEAVER, F. A., TORKELSON, A. R., ZYGMUNT, W. A. & BROWDER, H. P. (1978) Tissue culture cytotoxicity assay for cyclophosphamide metabolites in rat body fluids. *J. Pharmacol. Sci.*, **67**, 1009.